# Fiberless, Multi-Channel fNIRS-EEG System Based on Silicon Photomultipliers: Towards Sensitive and Ecological Mapping of Brain Activity and Neurovascular Coupling †

**DOI:** 10.3390/s20102831

**Published:** 2020-05-16

**Authors:** Antonio Maria Chiarelli, David Perpetuini, Pierpaolo Croce, Giuseppe Greco, Leonardo Mistretta, Raimondo Rizzo, Vincenzo Vinciguerra, Mario Francesco Romeo, Filippo Zappasodi, Arcangelo Merla, Pier Giorgio Fallica, Günter Edlinger, Rupert Ortner, Giuseppe Costantino Giaconia

**Affiliations:** 1 Department of Neuroscience, Imaging and Clinical Sciences, Institute for Advanced Biomedical Technologies, University G. D’Annunzio of Chieti-Pescara, Via Luigi Polacchi 13, 66100 Chieti, Italy; david.perpetuini@unich.it (D.P.); pierpaolo.croce@unich.it (P.C.); f.zappasodi@unich.it (F.Z.); arcangelo.merla@unich.it (A.M.); 2 Department of Energy, Engineering and Mathematical Models, University of Palermo, Viale delle Scienze 9, 90128 Palermo, Italy; giuseppe.greco17@unipa.it (G.G.); leonardo.mistretta@unipa.it (L.M.); raimondo.rizzo@unipa.it (R.R.); costantino.giaconia@unipa.it (G.C.G.); 3 ADG R&D, STMicroelectronics s.r.l., Stradale Primosole 50, 95121 Catania, Italy; vincenzo.vinciguerra@st.com (V.V.); mario.romeo@st.com (M.F.R.); piero.fallica@gmail.com (P.G.F.); 4 Guger Technologies OG, Herbersteinstrasse 60, 8020 Graz, Austria; edlinger@gtec.at; 5 g.tec Medical Engineering Spain S.L., Calle Plom 5-7, 08038 Barcelona, Spain; ortner@gtec.at

**Keywords:** functional near infrared spectroscopy (fNIRS), electroencephalography (EEG), multimodal neuroimaging, silicon photomultipliers, neurovascular coupling, clinical brain monitoring

## Abstract

Portable neuroimaging technologies can be employed for long-term monitoring of neurophysiological and neuropathological states. Functional Near-Infrared Spectroscopy (fNIRS) and Electroencephalography (EEG) are highly suited for such a purpose. Their multimodal integration allows the evaluation of hemodynamic and electrical brain activity together with neurovascular coupling. An innovative fNIRS-EEG system is here presented. The system integrated a novel continuous-wave fNIRS component and a modified commercial EEG device. fNIRS probing relied on fiberless technology based on light emitting diodes and silicon photomultipliers (SiPMs). SiPMs are sensitive semiconductor detectors, whose large detection area maximizes photon harvesting from the scalp and overcomes limitations of fiberless technology. To optimize the signal-to-noise ratio and avoid fNIRS-EEG interference, a digital lock-in was implemented for fNIRS signal acquisition. A benchtop characterization of the fNIRS component showed its high performances with a noise equivalent power below 1 pW. Moreover, the fNIRS-EEG device was tested in vivo during tasks stimulating visual, motor and pre-frontal cortices. Finally, the capabilities to perform ecological recordings were assessed in clinical settings on one Alzheimer’s Disease patient during long-lasting cognitive tests. The system can pave the way to portable technologies for accurate evaluation of multimodal brain activity, allowing their extensive employment in ecological environments and clinical practice.

## 1. Introduction

Investigation of brain function is becoming increasingly important in studying neurophysiological and neuropathological status. Multiple technologies have been developed to study brain signals coming from distinct neurophysiological mechanisms. In this perspective, it is fundamental to provide simultaneous multimodal brain monitoring able to record the different physiological facets of brain activity. Among multimodal neuroimaging, the integration of functional near-infrared spectroscopy (fNIRS) and electroencephalography (EEG) is showing promising capabilities [1].

EEG is an established technique that evaluates brain electrical activity with very high temporal resolution through measurements of scalp voltages originated by simultaneous activation of a large number of cortical neurons. EEG systems are widely used in research and clinical settings to monitor brain function and to diagnose brain dysfunction [2,3].

fNIRS is an optical technique that injects into the scalp, and measures from the same surface, near-infrared light (NIR, 650–950 nm of wavelength) that travels through the brain structures, historically relying on optical fibers for light delivering and collection. fNIRS estimates changes in the concentration of oxy-hemoglobin (O_2_Hb) and deoxy-hemoglobin (HHb) in the cortical layers exploiting their different NIR absorption spectra [4]. Brain activity can be inferred since these hemodynamic fluctuations are related to the underlying neural activity through the neurovascular coupling phenomenon [5,6]. fNIRS has different advantages when compared to other brain imaging modalities that measure brain hemodynamics (i.e., functional Magnetic Resonance Imaging (fMRI) and Positron Emission Tomography (PET)). It generally relies on cheap hardware, does not impose major physical constraints, and does not involve exposure to high magnetic fields and ionizing radiations. Moreover, in similarity with EEG, fNIRS is robust against motion artifacts, allowing brain activity investigation in heterogeneous populations (e.g., neonates, children, epileptic and Alzheimer Disease patients) and different experimental conditions (e.g., bedside, ambulatory settings, ecological environments) [7,8,9,10].

fNIRS systems can be classified in three main classes: Time-Domain (TD) [11], Frequency-Domain (FD) [12] and Continuous Wave (CW) [13] technology. CW systems rely on the simplest and cheapest optical technology and they measure the time-constant, i.e., CW, injected light that propagated within the head and the brain tissues. CW systems can provide estimates of hemoglobin oscillations over time but, because of the lack of photons time-of-flight information within the diffusive structures, they cannot provide a separate estimation of tissue absolute optical properties (i.e., absorption and reduced scattering coefficients) impairing the evaluation of hemoglobin concentration at baseline. However, since neurovascular coupling can be inferred from hemoglobin modulations, CW are the most common systems for brain monitoring in neuroscience research.

fNIRS did not find widespread employment in clinical practice [14]. This can be explained by the following current limitations:

- Interpretation of fNIRS results is not direct given the multiple physiological origins of the hemodynamic modulations [15].
- The presence of a large intersubject variability in the healthy population impairs the detection of a modified hemodynamic response in pathological conditions.
- fNIRS measures are sensitive to optical phenomena occurring within small volumes (of lateral dimension analogous to the source-detector distance) that have the shape of curved spindles (“bananas”); by changing the distance between the source and detector, different depth sensitivities can be obtained. These characteristics, potentially providing a better spatial and depth resolution than EEG, require many overlapping channels with high optode density to obtain a large field of view and spatially resolved brain monitoring, making standard sparse fNIRS systems not appropriate.
- The sensitive fiber-based technology is mechanically bulky whereas fiberless technology does not utilize sensitive detectors, often restricting fiberless fNIRS measurements to hair-free regions, such as the forehead, using a fixed and small interoptode (source-detector) distance [16,17,18,19], severely limiting field of view and depth investigation capabilities of the recordings.
- Integration of fNIRS with EEG is not common in clinical settings.

Whereas the first two drawbacks are intrinsic to the fNIRS methodology and require further investigation, the last three limitations can be solved by using sensitive fiberless technology (i.e., by placing sources and sensitive detectors directly on the scalp). Fiberless fNIRS avoids the use of bulky optical fibers through wearable probing while preserving the sensitivity that can be achieved with fiber-based instrumentation; moreover fiberless recording modality allows simple integration with EEG [20,21]. However, state-of-the-art fiberless CW-fNIRS systems generally employ photodiodes for light detection which provide light sensitivities that are orders of magnitude lower than detectors used in fiber-based technology (e.g., PhotoMultiplier Tubes, PMTs). Recently, highly sensitive solid-state detectors, such as single-photon avalanche diode (SPADs), have been employed for scalp-located recordings in fiberless fNIRS but this solution is not optimal since their detection area is very small, drastically reducing photon harvesting from the scalp.

In the last years, silicon photomultipliers (SiPMs), originally developed as single photon detectors in high energy physics [22], have been proposed for biomedical applications [23,24], including fNIRS [25,26,27,28,29,30]. SiPMs are bi-dimensional arrays of pixelated photodetectors made of SPADs each acting as a single photon counter [31]. The matrix geometry of SiPMs ensures a large detection area (range of 1–20 mm^2^) for maximized photon harvesting from the scalp. Compared to other semiconductor photodetectors, SiPMs present major advantages of sensitivity, high internal gain, and speed of response [23]. SiPMs provide a similar responsivity to PMTs which is ~3 to ~5 orders of magnitude higher than photodiodes or avalanche photodiodes (APDs). With respect to PMTs, SiPMs exploit the advantages of semiconductor technology being compact, easy to handle, mechanically robust, optically resistant, electrically reliable and operating at low voltage. Previous work in fNIRS applications demonstrated that SiPMs can reach light sensitivities of few femtoWatts (fW) [32,33]. Considering injectable light source power, average light attenuation within the head, and SiPMs detection area, this sensitivity allows to perform fNIRS measurements at 70–80 mm of interoptode distance. These distances can enable the investigation of brain regions up to a depth of ~30 mm from the scalp and to utilize SiPMs in multidistance high density optical arrays for tomographic approaches (i.e., Diffuse Optical Tomography, DOT) using fiberless wearable fNIRS technology [34,35], and with possible integration with EEG.

Development of a novel fiberless fNIRS-EEG system is here presented. The device was constituted of a multi-channel, multi-distance, SiPM-based CW-fNIRS component and a modified version of a commercial EEG instrumentation (g.HIamp, g.tec medical engineering). The system was developed within an H2020 project funded by the European Union, termed ASTONISH (Advancing Smart Optical Imaging and Sensing for Health), with the aim to concurrently measure brain hemodynamic and electrical activity, as well as their link, i.e., the neurovascular coupling, in clinical settings. The fNIRS component was made of 16 bicolor light emitting diodes (LEDs at 735 nm and 850 nm of wavelength) and 16 SiPMs, for a total of 256 (16 × 16) channels; the EEG instrumentation was constituted of 19 electrodes. Both the fNIRS and EEG components could be easily expanded up to 32 bicolor LEDs and 32 SiPMs for a total of 1024 (32 × 32) channels and 256 EEG electrodes. The LEDs and SiPMs were encapsulated in polymeric holders with dimensions and shapes resembling EEG electrodes. These characteristics allowed the fNIRS optical probes and the EEG electrodes to be held on the scalp through the same commercial EEG cap. fNIRS analog signals were sent to and collected from the scalp through thin electric cables allowing to overcome standard mechanical constraints introduced by optical fibers.

All the required fNIRS electronic processing was hosted by a Zynq Field-Programmable Gate Array (FPGA)-based System on Chip (SoC), featuring an adjustable LED current and configurable SiPM gain. This architecture was already presented in [36]. The FPGA implemented a full digital lock-in in order to minimize ambient light contamination, electronic noise, and interference with the EEG. The fNIRS and EEG components were effectively synchronized with the fNIRS acting as a slave system.

In order to characterize the fNIRS component performances, the system noise equivalent power (NEP) was estimated in an anechoic chamber. Furthermore, other key features of CW-fNIRS, namely linearity between impinging light on the detector and measured signal as well as channels’ crosstalk, were characterized on optical phantoms. Moreover, in vivo recordings were performed on several subjects, evaluating hemodynamic and electrical brain activity in visual, motor and pre-frontal cortices during a variety of tasks inducing brain response in the investigated cortical area. Finally, the system was tested in an ambulatory setting for ecological monitoring of brain activity in one early Alzheimer’s Disease (AD) patient during long-lasting (~1 h) cognitive test routinely performed for AD diagnosis.

## 2. Materials and Methods

### 2.1. fNIRS Instrumentation: Silicon Photomultipliers and Light Emitting Diodes

SiPMs are semiconductor light detectors fabricated in silicon planar technology. Their structure is made of a bi-dimensional array of SPADs acting as photon counters. The SPAD junction is slightly biased through an overvoltage of about 10% of the breakdown voltage (~27 V), and it is able to stay quiescent (i.e., no current is generated) for a relatively long period of time (a fraction of a second). When a photon is absorbed in the depletion volume, primary charge carriers (electrons and holes) enter the high field region of the depleted volume and trigger the avalanche multiplication process. The fast-leading edge of the corresponding current pulse can be used for detection and timing of the photo-generated carriers. In a SiPM, the SPADs are connected to the power supply through an integrated quenching resistor which turns off the avalanche current and resets the activated photodiodes. Since the pixels are short circuited, the amount of charge collected at the SiPM output is given by the superposition of the binary signals produced, which in turn creates an analog signal proportional to the number of incident photons. Therefore, SiPMs behave as analogue devices able to detect weak photon fluxes.

Two types of structures are used in SiPMs: p-on-n or n-on-p configurations. For CW-fNIRS application, n-on-p configuration is more suitable because of its higher photon detection efficiency in NIR when compared to the p-on-n version of the technology [37].

**Figure 1:**
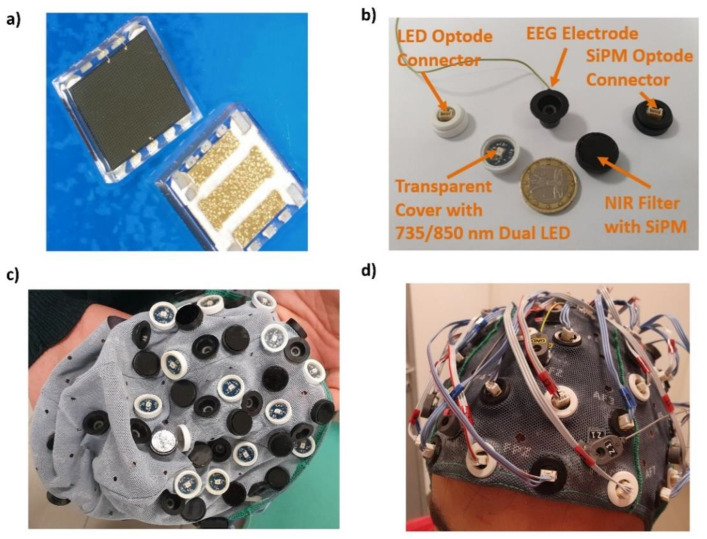
(**a**) Front and back view of a large area n-on-p silicon photomultiplier (SiPM) manufactured at STMicroelectronics clean room facilities; (**b**) SiPMs and LEDs encapsulated in black and white cases respectively, together with an electroencephalography (EEG) electrode and a 1 Euro coin; (**c**) Functional Near-Infrared Spectroscopy (fNIRS) probes and EEG electrodes located onto a commercial EEG cap; (**d**) EEG-fNIRS probing located on a subject’s head during in vivo measurement.

N-on-p SiPMs manufactured at STMicroelectronics clean room facilities were employed in the system developed (Figure 1a). The devices were fabricated on p-type, heavily doped, silicon substrates. A thin p-type epitaxial layer was grown on the wafer to form the diode anode. A heavily doped, very thin, n-type polysilicon layer was added by a high temperature Chemical Vapor Deposition. The SPAD diodes had a n++/p-/p+ doping profile. The quenching resistor, made of low-doped polysilicon, was integrated inside the cell. Thin optical trenches, filled with oxide and tungsten, surrounded the active area to reduce crosstalk between adjacent microcells. The performances of these SiPMs are comparable to those of similar technologies available on the market in terms of gain, photon detection efficiency and dark noise rate. SiPMs had a large area of 4.18 × 4.68 mm^2^ and 4871 square microcells with 60 µm pitch. The detection area, coupled with a geometrical fill factor of 67.4%, maximized photon harvesting from the scalp. Epitex bicolor SMT735 AlGaAs LEDs (emitting at 735 nm and 850 nm) in SMD package were employed as light sources. The LEDs had an area of 2.6 × 4.5 mm^2^, viewing angle of 55°, average spectral bandwidth of 20 nm at 735 nm and 35 nm at 850 nm wavelength. The power emission of these LEDs ranged between 1 and 20 mW.

### 2.2. fNIRS Instrumentation: Optical Probes

Each SiPM was mounted on a small printed circuit board (PCB) of ~100 mm^2^ section, containing the detector and passive components (a 1 kΩ resistor and a 100 nF capacitor). Each bicolor LED was mounted on a PCB of the same size. The boards were equipped with plug-in connectors on the back and inserted (glued) into small plastic cases (white for LEDs, black for SiPMs) (Figure 1b). The SiPM case housed a NIR long-pass filter with a refraction index of 1.5, 700 nm cut-off wavelength and an optical transmission higher than 90% in NIR range. The use of the filter reduced ambient light contamination and SiPM dark current resulting in a higher signal-to-noise ratio (SNR) in a wide range of operation conditions and biases. The optical cases were held on the scalp, together with the EEG electrodes (Figure 1b), by a commercial EEG cap (Figure 1c,d). The electrical connection to the acquisition electronics was made of flat cables 2 m long.

### 2.3. fNIRS Instrumentation: Architecture

**Figure 2:**
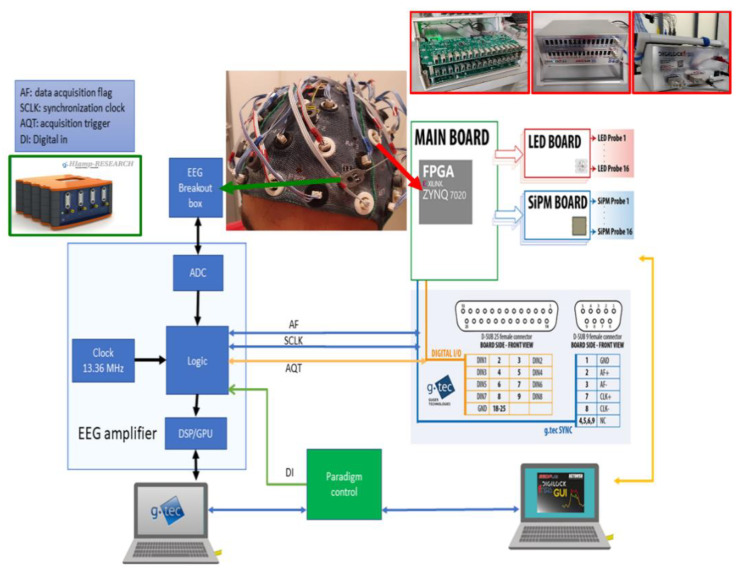
fNIRS-EEG system architecture and boxes enclosing the electronic components.

The fNIRS electronic architecture was designed based on a digital lock-in, namely the dual-phase lock-in amplification technique (performed at 1562.4 Hz modulation frequency). Notice that, a lock-in modulation frequency of 1562.4 Hz, when considering 16 bi-color LEDs (32 time bins), allows a maximum time-multiplexed measurement rate of 48.825 Hz. Details of the procedure and architecture can be found elsewhere [36,38]. The fNIRS system, named DigiLock, was constituted of three custom boards (analog to digital converter (ADC), LED and SiPM boards) and a FPGA board (Figure 2). The ADC board used two sigma-delta converters (TI ADS1298). The LED board implemented an adjustable current and 32 time-multiplexed outputs controlling the 16 bicolor LEDs. The SiPM board hosted an adjustable DC\DC converter for the SiPM bias generation and a protection circuitry against overvoltage and overcurrent. Importantly, each combination of LED current and SiPM bias could be adjusted during the time-multiplexing cycle to optimize signal acquisition for each LED and SiPM couple. The FPGA board (MYIR Z-turn) was a Single Board Computer built around the Xilinx Zynq 7Z020 All Programmable SoC. The Xilinx Zynq family integrates a Xilinx 7-series FPGA with a hardwired dual-core ARM Cortex-A9. In addition to the Zynq SoC, the board was equipped with RAM and FLASH memory, USB, Ethernet and other peripherals. The lock-in algorithm was implemented within the FPGA with a low-level hardware description language (HDL). The FPGA handled the synchronization among the LEDs, the ADCs and the lock-in elaboration chain. Automated calibration of each LED current and SiPM bias couple was implemented employing a procedure based on the identification of the heartbeat periodicity [39]. After data acquisition, the processors formed a custom data packet that was sent to the host PC through an Ethernet link. The host PC collected the packets and generated an output file in Hierarchical Data Format (HDF5). All the boards were enclosed in a small case (240 × 180 × 120 mm^3^), with the probe connectors on the front panel and power and communication connectors on the back panel (Figure 2).

To synchronize fNIRS Digilock and the EEG instrumentation, a handshake protocol was implemented with start and acknowledge signals between the two machines (Figure 2). EEG was configured as a master, whereas the fNIRS component acted as a slave. The digital lines were embedded in a Centronics cable.

### 2.4. EEG Instrumentation

The EEG component of the system was a modified version of a commercially available biosignal amplifier (Figure 2, g.HIamp, g.tec medical engineering). The amplifier was able to acquire up to 256 channels with a resolution of 24 bits and with the possibility to measure, together with EEG signals, electrooculographic, electromyographic and electrocardiographic signals. The sampling frequency could be adjusted in the range of 256–4800 Hz. The device delivered an impedance check unit. Active EEG electrodes with 2 pin safety connectors were used via multichannel breakout connector boxes (64 channels per box). A digital input was available to acquire trigger signal from any external device. The EEG component could be controlled with an Application Programming Interface in MATLAB/Simulink or C-API.

### 2.5. fNIRS Instrumentation: Noise Equivalent Power Evaluation

The method reported in Wiser et al. [40] was used to evaluate the NEP of the fNIRS component.

In order to avoid any electromagnetic contaminations, the fNIRS component was placed within an anechoic chamber, located at University of Palermo premises. This facility features noisy-free power supply and ethernet connection chain, giving the possibility to leave the controlling PC outside the chamber. Under these deep dark conditions, measurement trials were performed by using the fNIRS component equipped with one SiPM, without any LED sources and with a sampling frequency of 781.2 Hz.

A preliminary test was performed, positively verifying that, by short circuiting the input where usually SiPM is located, noise level of the fNIRS instrument was well below the one collected when SiPM was connected and power supplied.

Moreover, a further test was performed to find the minimum time interval in order to obtain a stable voltage and current measures. This test yielded a signal variability below 1% when the recording time exceeded 25 s from the start of the SiPM voltage supply. Given these results, the recording period for each trial lasted 300 s providing stable estimates of the photocurrent due to noise.

The current noise level (expressed in mA) was estimated for several working modalities of the SiPM from under- to over-threshold supply voltages (from 26 up to 29 V). By knowing the responsivity (expressed in mA/W) of the SiPMs employed [41], the current was transformed into optical power by dividing the measured noise current by the responsivity of the SIPM for the given wavelength and supply voltage. Since NEP is the optical power correspondent to a SNR of 1 (0 dB), the mentioned estimate provided an end-to-end NEP for the fNIRS component in the working conditions used.

### 2.6. fNIRS Instrumentation: Phantom Validation

fNIRS instrumentation linearity and channels’ crosstalk were assessed, at a measurement sampling rate of 48.825 Hz, on a silicone phantom mimicking biological tissue optical property (absorption coefficient μ_a_ = 0.0106 mm^−1^, reduced scattering coefficient μ_s_’ = 0.95 mm^−1^ at 735 nm, μ_a_ = 0.0103 mm^−1^, μ_s_’ = 0.90 mm^−1^ at 850 nm, refractive index η = 1.4 at both wavelengths). The system linearity to impinging light was evaluated by exploiting the exponential decay of back-reflected light, and hence recordings SNR, as a function of distance in a homogeneous diffusive medium (i.e., optical phantom). A LED was moved every 10 s, 10 mm further from a SiPM with distances in the range of 30−70 mm; the procedure was iterated for 16 randomly selected couples of SiPM and bicolor LED. The system channels crosstalk was instead quantified by locating a SiPM and a bicolor LED at 30 mm distance and by sinusoidally moving the LED at a frequency of 0.33 Hz between 30 and 50 mm for ~1 min. The other 15 SiPMs were positioned on a control phantom in a light isolated box; the procedure was iterated for 16 randomly selected couples of SiPM and bicolor LED. Interference was estimated by inspecting the signals coming from the SiPMs on the measurement and control phantoms.

### 2.7. fNIRS-EEG System: In Vivo Validation

**Figure 3:**
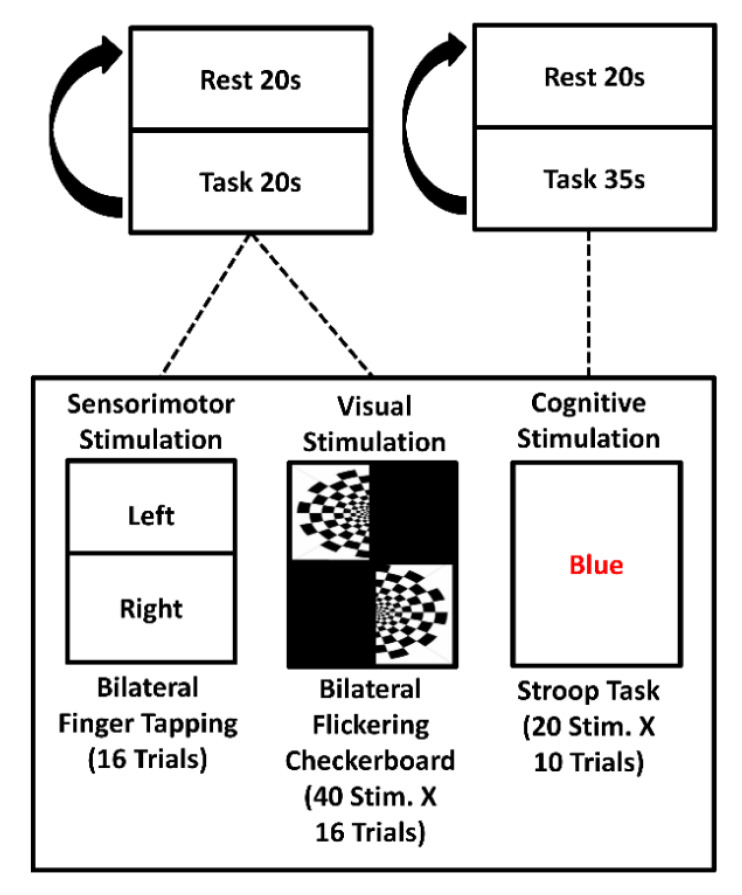
Battery of tasks performed for the in vivo validation of the instrument.

In vivo validation was performed in agreement with the ethical standards of Helsinki Declaration, 1964, and it was approved by the local Human Board Review and Ethical Committee. Seven informed healthy subjects (all males, mean age: 30 ± 6 years) set comfortably on a chair and performed a battery of block-designed tasks inducing brain activity in the motor, visual and prefrontal cortices (Figure 3).

To elicit motor cortex activity, the subjects were requested to perform a left-hand and right-hand finger tapping task with the instruction (‘left’, ‘right’ and ‘stop’ commands) presented on a 19′ screen. The experiment was composed of 20 s of rest alternated with 20 s of task repeated 8 times for each of the two stimuli (left or right finger tapping, presented in a pseudorandom order), for a total of 16 trials. To induce visual cortex activity, the subjects were requested to stare at the 19′ screen at a distance of ~90 cm and to look at a fixation cross while left and right field-of-view flickering black-and-white checkerboards were presented at a frequency of 2 Hz [42]. The task was composed of 20 s of rest and 20 s of stimulation for 8 trials repeated 8 times for each of the two stimuli (left or right visual hemifield, presented in a pseudorandom order), for a total of 16 trials. To elicit frontal cortex activity, a Stroop task was performed [43]. The Stroop task is a paradigm that involves the inhibition of automatic responses. Stimulus conflict-words and color patches were used; the participants were requested to press a button associated to the color of the letters presented (4 colors employed: Red, Blue, Yellow, Green). The inhibition of automatic response occurs when there is a mismatch between the name of a color and the color that is printed on the screen (i.e., the word ‘red’ printed in blue ink instead of red ink). The experiment was made of 10 trials of 20 stimulations with a maximum interstimulus time interval of 2 s. All the stimulations were presented using Psychtoolbox, a software implemented in Matlab environment [44].

The synchronization with the recording instrumentation was obtained with a photosensor positioned on the stimulation screen that was activated by changes in screen luminosity.

**Figure 4:**
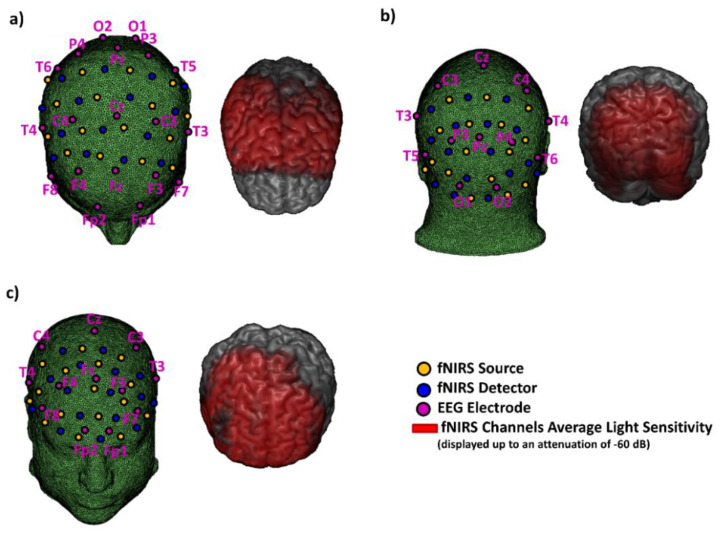
fNIRS optodes and EEG electrodes configurations employed during the administration of the different tasks. fNIRS channels layout varied according to the task investigating the motor (**a**), visual (**b**), and prefrontal cortices (**c**). The electrodes were located according to the 10/20 system. All three recordings were performed employing 16 bicolor LEDs, 16 SiPMs and 19 electrodes. Light sensitivity was computed with the Finite Element Method (FEM) [46] using a template head and brain.

fNIRS optodes configuration varied according to the task in order to investigate the cortical area involved (Figure 4). The recordings were performed employing all the optical probes available (16 SiPMs and 16 bicolor LEDs). The channels’ interoptode distances, derived from the optodes configurations, varied between 30 and 50 mm; the distances were adequate to reach the cortical surface with a high SNR [45]. Light sensitivity for the different configurations was assessed through Finite Element Method (FEM), performed on a template brain (Figure 4) [46]. fNIRS data were acquired at 48.825 Hz of measurement sampling rate.

EEG recordings were performed employing 19 electrodes. The electrodes were placed according to the 10/20 International System (Fp1, F3, F7, C3, T3, P3, P7, O1, Fz, Cz, Pz, Fp2, F4, F8, C4, T4, P4, P8, O2, Figure 4). The right earlobe was used as reference. Data were sampled at 512 Hz.

Using the optodes configuration employed in the Stroop task, the prototype was finally tested in a clinical environment on 1 informed Alzheimer Disease (AD) patient (male, 75 years old) during a long (~1 h) battery of cognitive tests performed for early AD diagnosis [47]. The ecological measurements were conducted within an ambulatory setting and without altering clinical practice. The patients sat in front of the doctor and the examination was performed through a free doctor-patient interaction.

### 2.8. fNIRS-EEG Data Analysis

For the fNIRS data analysis, the raw signals for each of the two wavelengths were converted into optical densities (ODs) according to the equation:

(1) $$\mathrm{OD}\;=-\ln\left(I\left(t\right)/I_{o}\right)$$

where I (t) is the time dependence of the recorded signal intensity and $I_{o}$ is its initial value. Raw signal and ODs were inspected based on their spectrum and continuous wavelet transform. For further analysis of in vivo measurements, a movement artifact correction procedure was applied to the ODs [48]. Moreover, a 0.01−0.2 Hz band-pass, zero-lag, 5th order Butterworth digital filter was applied to ODs to highlight brain hemodynamics. Changes in the concentration of O_2_Hb and HHb were derived from each channel based on the modified Lambert-Beer Law [49]:

(2) $$\left[\begin{array}{c} O_{2}\mathrm{Hb}\\ \mathrm{HHb} \end{array}\right]=\frac{1}{\rho }{\left[\begin{array}{cc} \varepsilon_{O_{2}\mathrm{Hb}}\left(\lambda_{1}\right)\cdot \mathrm{DPF}\left(\lambda_{1}\right)\; & \varepsilon_{\mathrm{HHb}}\left(\;\lambda_{1}\right)\cdot \mathrm{DPF}\left(\lambda_{1}\right)\\ \varepsilon_{O_{2}\mathrm{Hb}}\left(\lambda_{2}\right)\cdot \mathrm{DPF}\left(\lambda_{2}\right)\; & \varepsilon_{HHb}\left(\;\lambda_{2}\right)\cdot \mathrm{DPF}\left(\lambda_{2}\right) \end{array}\right]}^{-1}X\left[\begin{array}{c} \mathrm{OD}\left(\lambda_{1}\right)\\ \mathrm{OD}\left(\lambda_{2}\right) \end{array}\right]$$

where ρ is the interoptode distance, ε are the extinction coefficients for the two chromophores and the DPFs are the differential pathlength factors at the wavelengths of interest ($\lambda_{1}$ and $\lambda_{2}$). The extinction coefficients were extracted from Zijlstra et al. [50] (ε_O2Hb,735nm_ = 0.014 mm^−1^, ε_O2Hb,850nm_ = 0.025 mm^−1^, ε_HHb,735nm_ = 0.039 mm^−1^, ε_HHb,850nm_ = 0.019 mm^−1^). The DPFs were derived by Scholkmann and Wolf and Chiarelli et al. (DPF_735nm_ = 6, DPF_850nm_ = 5.5) [51,52].

For each of the three tasks (motor, visual and Stroop tasks), both a grand-average and a general linear model (GLM) approach [53,54] were employed for visualization and statistical inference about brain activation.

For the grand-average estimation, O_2_Hb and HHb responses in the different trials were averaged from 5 s prior to the task up to 15 s after the task and baseline corrected by subtracting the average hemoglobin concentration within the rest period.

In order to statistically quantify and to spatially identify brain activation, a GLM approach was employed. GLM is a standard statistical procedure for the evaluation of functional brain activity (either based on fNIRS or fMRI) [53]. This statistical analysis was conducted for both O_2_Hb and HHb. Single subject stimulation was modelled through a boxcar function that was convolved with a canonical hemodynamic response function (cHRF) to provide an ideal hemodynamic response to the task. GLM was performed for each subject and channel considering the ideal response as independent variable and the estimated hemoglobin oscillation as dependent variable. This analysis provided a β value, representing the strength of the activation, for each subject, channel, task and type of trial within the task. As a first level analysis, parametric t-tests were performed on βs to obtain a statistical map for each subject and channel for a defined contrast. Left vs. Right contrast was evaluated for motor and visual tasks to highlight the stronger contralateral activation in sensorimotor and visual regions. For the Stroop task, Stimulation vs. Rest contrast was evaluated. Group statistical maps of brain activity were computed from the single subject maps through a second level group analysis based on parametric t-tests. The group-level O_2_Hb and HHb t-scores were finally back-projected onto a template cortical surface relying on FEM [46].

For the EEG data analysis, saturated or corrupted epochs were firstly rejected based on visual inspection. An automatic procedure based on Independent Component Analysis was applied to identify and to remove cardiac and ocular artifacts, as well as signals coming from muscle activity [55,56]. For the motor and visual tasks, data were re-referenced to the Fz electrode whereas for Stroop task a common average reference was used. Data were then band-pass filtered between 0.1 and 80 Hz with a 2nd order, zero-lag, Butterworth digital filter.

Brain activity during the finger tapping task was estimated through an Event Related Desynchronization (ERD) analysis. Power Spectral Densities (PSDs) were calculated during rest and motor execution by means of Welch’s method (2 s intervals, frequency resolution of 0.5 Hz). Band powers in the alpha and beta bands were obtained as average PSD in 8–12 Hz and 15–25 Hz frequency ranges. ERD was defined as:

(3) $$\mathrm{ERD}\;=100\;\frac{P_{T}-{\;P}_{R}}{P_{R}}$$

where P_T_ is the band power during the task and P_R_ is the band power during rest. Visual Evoked Potentials (VEPs) were estimated to evaluate electrical brain activity during the visual stimulation. To retrieve VEPs, data were segmented into epochs from 50 ms before to 300 ms after the onset of the pattern-reversal stimulation and averaged. According to standard procedures [57], the N75, P100 and N135 components were identified on O1 and O2 electrodes. The amplitude of P100 was evaluated from the preceding N75 peak. A similar procedure was performed to obtain Event Related Potentials (ERPs) induced by the Stroop task: response to single stimulations were averaged in windows from 50 ms before to 600 ms after the stimulus onset. The N200 component on Fz electrode was evaluated [58]. Group analyses on ERDs, VEPs and ERPs were employed on average single subject values.

A final analysis was conducted to evaluate the ability of the developed system to assess neurovascular coupling. Specifically, subject’s statistical dependence between the strength of the EEG and fNIRS brain activity was estimated. The EEG-fNIRS coupling was assessed during the motor and visual tasks, where EEG and fNIRS inferred brain activity are known to be spatially consistent and exploited the contralateral organization of the motor and visual cortices. The difference between right and left channels of the Left vs. Right contrast was estimated for both EEG and fNIRS O_2_Hb in each subject and a linear regression of the results among all subjects was performed. The intensity of the electrical brain activity was estimated based on subject’s beta band ERD for the motor task and as P100 for the visual stimulation. The intensity of the hemodynamic brain activity was estimated based on O_2_Hb t-scores derived from the GLM analysis. Electrode and optode couples, located onto C3 and C4 and onto O1 and O2 (10/20 system), were selected for the motor and visual tasks, respectively.

## 3. Results

### 3.1. fNIRS System: Noise Equivalent Power Evaluation

**Figure 5:**
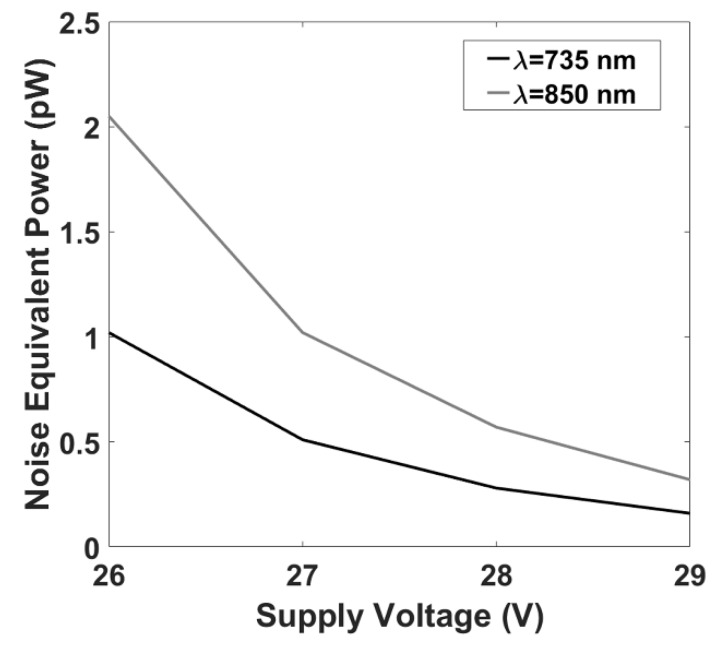
fNIRS component end-to-end noise equivalent power (NEP) as a function of SiPM supply voltage.

Figure 5 reports the end-to-end NEP at different SIPM supply voltages at the two wavelengths employed. Supply voltage was evaluated from 26 V, which is below its breakdown voltage, up to 29 V, which is beyond the breakdown voltage. The NEPs were evaluated at a sampling frequency of 781.2 Hz. Although SiPMs usually feature higher responsivities for overvoltages exceeding 29 V, they generally lose response linearity, which is essential for fNIRS. For this reason, these voltages were not characterized.

NEP values were below 1 pW when SiPM was supplied with a voltage above the breakdown. These results would allow the collection of fNIRS signals with a good SNR at a distance well beyond 50 mm [40], also considering the large detection area of the detector, the high sampling frequency used for these tests and the physiological bandwidth of fNIRS that does not exceed the fraction of Hz.

### 3.2. fNIRS System: Phantom Validation

**Figure 6:**
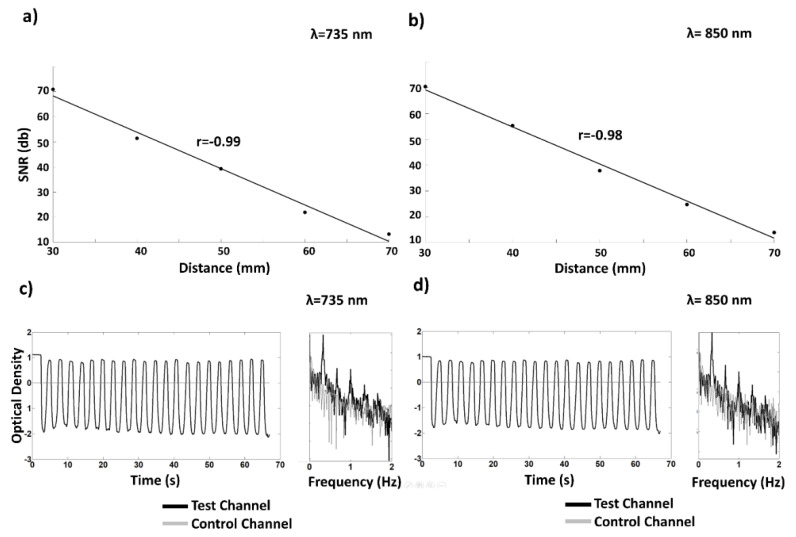
(**a**) Example of the decay with distance of the log signal-to-noise ratio (SNR) (dB), when SiPM voltage was optimized at 30 mm interoptode distance, for a randomly selected SiPM and LED couple at 735 nm; (**b**) example of the decay with distance of the log SNR (dB) for a randomly selected SiPM and LED couple at 850 nm; (**c**) example of temporal oscillations in optical densities (ODs) and associated spectra obtained from crosstalk measurements at 735 nm. The induced oscillation in the test channel was absent in the control channel; (**d**) example of temporal oscillations in ODs and associated spectra obtained from crosstalk measurements at 850 nm. The induced oscillation in the test channel was absent in the control channel.

Figure 6a,b shows examples of the decay with distance of the log SNR of the electrical signal (dB), when SiPM overvoltage was optimized at 30 mm interoptode distance. Figure 6a shows the result obtained for a SiPM and LED at 735 nm whereas Figure 6b shows the result at 850 nm. Both wavelengths showed a strong linearity, with a log SNR correlation with distance of r = −0.99 and r = −0.98. For all the source-detector couples, correlation coefficients were always below −0.95. Importantly, linearity was held up to 70 mm interoptode distance showing the high sensitivity of the SiPMs.

Figure 6c,d shows examples of temporal oscillations in ODs and associated spectra obtained from the measurements assessing channels’ crosstalk. The modulation in the signal acquired in the test channel (where the interoptode distance was periodically varied) was clearly absent in the control channel (where the interoptode distance was kept constant). The same results were obtained for both wavelengths (Figure 6c,d) and for all the channels tested.

### 3.3. EEG-fNIRS System: In Vivo Validation

**Figure 7:**
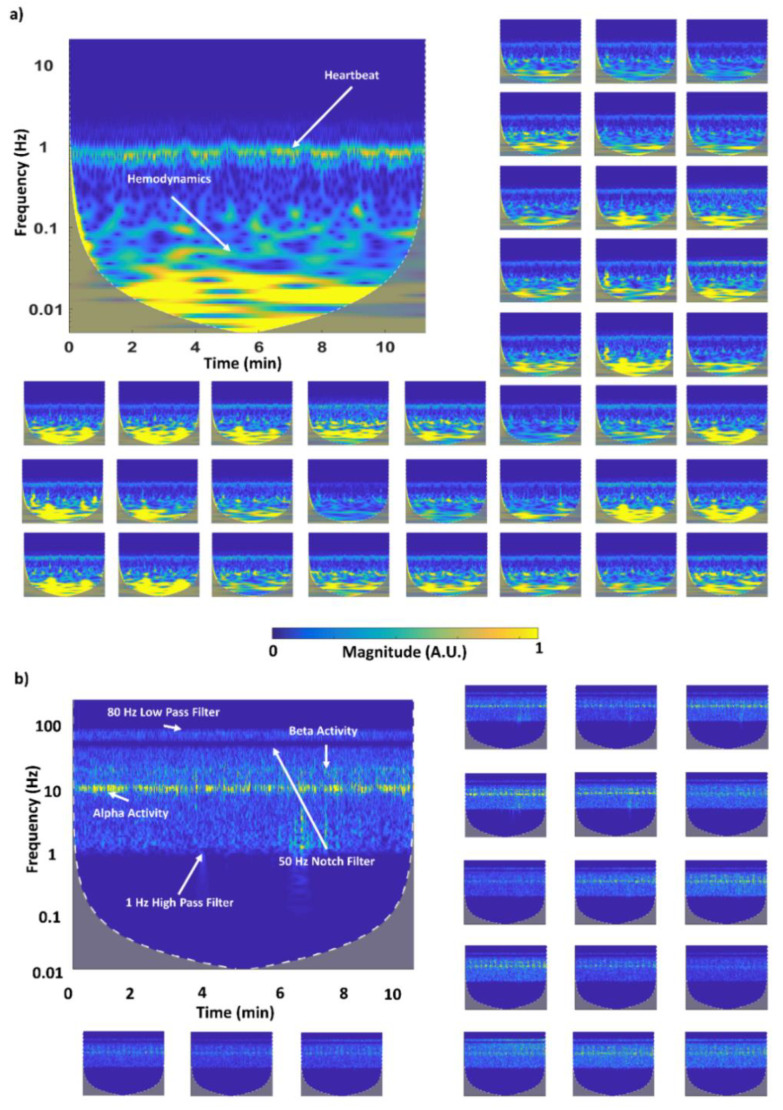
(**a**) Example of continuous wavelet decompositions of fNIRS ODs performed during the motor task in one subject. The recordings are reported for signals in multiple channels for the 850 nm wavelength. In the time-frequency decomposition, hemodynamic (below 0.1 Hz) and heartbeat components (at around 1 Hz) are visible in all the channels displayed. The presence of a heartbeat signal is considered indicative of a high SNR of continuous wave (CW)-fNIRS recordings; (**b**) example of continuous wavelet decompositions of EEG recordings performed during the motor task in one subject. A clear alpha band component (at around 10 Hz) is present in all the displayed channels, depicting the quality of the EEG recordings.

Figure 7a reports continuous wavelet decompositions of fNIRS ODs at 850 nm acquired in one subject performing the finger tapping task. Within the time-frequency decomposition plot, hemodynamic components are visible (below 0.1 Hz) together with a clear heartbeat main frequency component (at around 1 Hz) in all the channels displayed. The wavelength 850 nm is reported because of its higher sensitivity to the heartbeat modulation with respect to the 735 nm wavelength (high absorption of O_2_Hb, majorly present in pulsating arteries). A visible heartbeat in the CW-fNIRS recordings is considered highly indicative of a good SNR because heartbeat modulations in ODs are of the same order of magnitude of that induced by the neurovascular coupling phenomenon [39].

Figure 7b reports, for the same experiment and subject, continuous wavelet decompositions of the 19 EEG recordings. Typical EEG components are visible, with a strong 10 Hz (alpha band) modulation and, importantly, no EEG-fNIRS interference is present (e.g., presence of spikes caused by LEDs switching).

**Figure 8:**
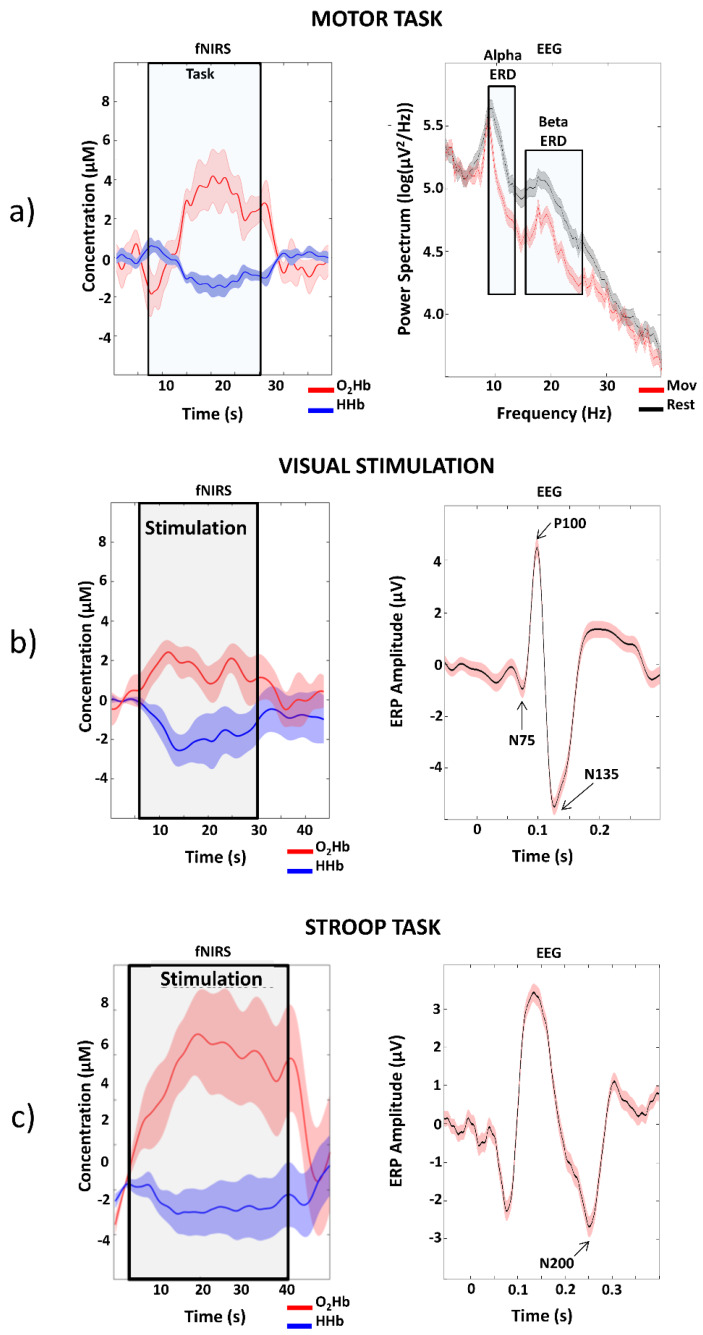
Examples of single channel block-average hemodynamic responses, and associated standard error, as measured by fNIRS together with Event Related Desynchronizations (ERDs), Visual Evoked Potentials (VEPs) and Event Related Potentials (ERPs) as measured by EEG during motor (**a**), visual (**b**) and Stroop (**c**) tasks.

Figure 8 reports examples of single subject, single channel, average hemodynamic responses as measured by fNIRS together with average electrical responses as measured by EEG for the three tasks performed. Typical hemodynamic activity is visible in the fNIRS signal, i.e., increase in O_2_Hb associated with a smaller decrease in HHb of few µM (Figure 8a–c). For the EEG, ERD (reduction in power) is visible in the alpha and in the beta bands during the motor task (Figure 8a) with respect to rest and typical VEPs (Figure 8b) and ERPs (Figure 8c) are present in response to the visual and Stroop tasks.

**Figure 9:**
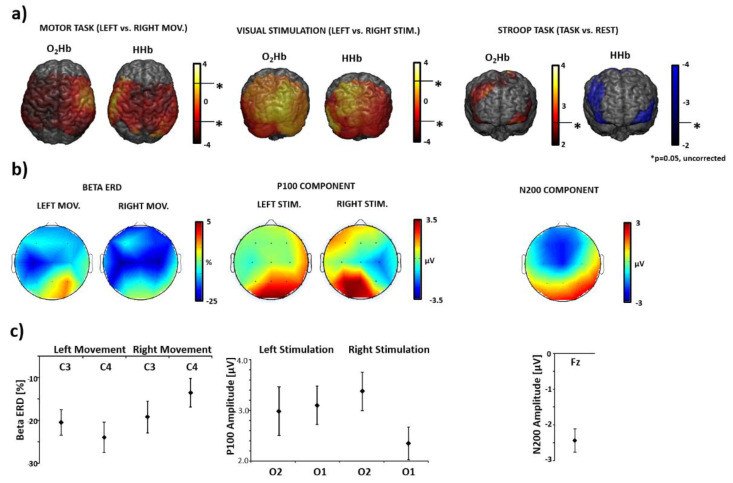
(**a**) fNIRS oxy-hemoglobin (O_2_Hb) and deoxy-hemoglobin (HHb) group level t-score maps obtained employing general linear model (GLM) analysis and back-projection onto a template brain for the motor (Left vs. Right contrast), visual (Left vs. Right contrast) and Stroop tasks (Task vs. Baseline); (**b**) EEG group level average topographies of beta ERD during finger tapping, P100 (mean latency: 106 ± 9 ms) during the visual stimulation and N200 ERP for the Stroop task; (**c**) group level average, and associated standard error, ERD in C3 and C4 electrodes for the left and right finger tapping task, group level average, and associated standard error, of P100 in O1 and O2 electrodes for the left and right visual stimulation and group level average, and associated standard error, of the N200 in Fz for the Stroop task (* *p* = 0.05, uncorrected).

Figure 9a reports fNIRS t-score maps obtained employing a first level GLM analysis and second group level t-test analysis. Stronger activations (i.e., increase in O_2_Hb and decrease in HHb) in the contralateral motor and visual cortices were found when evaluating the Left vs. Right contrast. For the Stroop task, brain activity (i.e., increase in O_2_Hb and decrease in HHb during the task) was identified in frontal regions which are compatible with those found in previous work (lateral prefrontal cortices [59]). Two-tailed, uncorrected 5% null hypothesis thresholds (*p* = 0.05) are reported on the color bars. Figure 9b reports EEG group level average topographies of visual P100 component (mean latency: 106 ± 9 ms) and beta ERD; P100 and beta ERD showed maximum signal in the controlateral visual (O1 for right visual stimulation, O2 for left stimulation) and motor areas (C3 for right movement and C4 for left movement). Figure 9b also reports EEG group level average topography of the N200 ERP for the Stroop task. A clear negative component in the frontal regions was found in accord with the literature [60]. Figure 9c reports group level average ERD in C3 and C4 electrodes for the left and right finger tapping task, group-level average P100 component in O1 and O2 electrodes for the left and right visual stimulation and group level average N200 in Fz for the Stroop task, further corroborating the found activation patterns that met the expectations.

**Figure 10:**
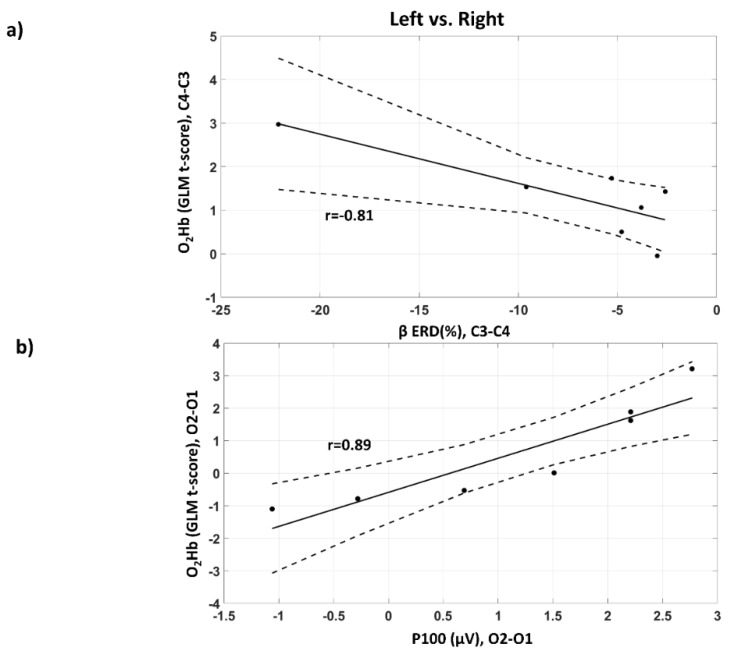
Among subjects’ linear association between EEG electrical and fNIRS hemodynamic brain activity. The strength of the contralateral activation (Left vs. Right) in these cortices after lateralized stimulation is evaluated in the (**a**) motor cortex (C4-C3, 10/20 system), and in the (**b**) visual cortex (O2-O1, 10–20 system). Regression lines and associated confidence intervals (CIs) are reported.

Figure 10 reports the evaluation of the link between EEG electrical and fNIRS hemodynamic brain activity. The regression lines together with their confidence intervals are shown [61]. The controlaterality of brain activity (Left vs. Right contrast) was evaluated in motor (Figure 10a) and visual (Figure 10b) cortices (C4-C3 and O2-O1, 10/20 locations) for both EEG and fNIRS. A significant correlation in EEG and fNIRS controlaterality extent was obtained for both motor (r = −0.81, df = 5, *p* = 0.02) and visual tasks (r = 0.89, df = 5, *p* = 6 × 10^−3^) depicting a neurovascular coupling effect. Importantly, as expected, for the motor task the correlation was negative (increased O_2_Hb GLM t-score coupled with stronger EEG desynchronization in the beta band) whereas for the visual stimulation the correlation was positive (increased O_2_Hb GLM t-score coupled with higher P100 amplitude). Notably, whereas the correlation of the visual stimulation is highly robust, the correlation obtained for the motor task, although significantly different from 0, might be driven by a single outlier.

### 3.4. Proof of Concept: Ecological EEG-fNIRS in One Alzheimer Disease Patient during Cognitive Clinical Tests in an Ambulatory Environment

**Figure 11:**
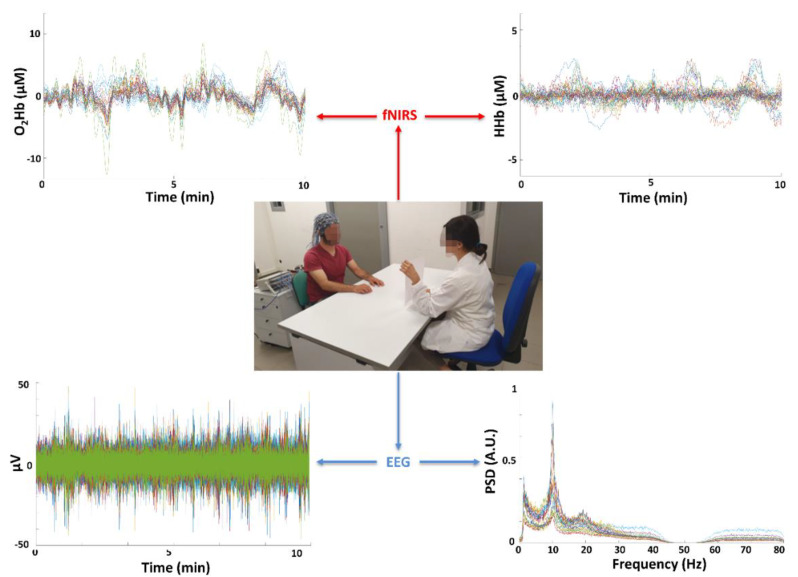
Example of ecological fNIRS-EEG recordings (10 min zoom) performed in clinical settings with the developed instrument in 1 Alzheimer’s Disease (AD) patient during cognitive tests lasting ~1 h. The tests are routinely utilized for early AD diagnosis. The patient was free to interact with the doctor and the probing did not impose any major physical constraints. The high quality of the data and absence of significant artifacts for both fNIRS and EEG are visible.

Figure 11 shows an ecological recording (10 min zoom) performed in clinical ambulatory settings through the developed instrumentation, in one AD patient measured during ~1 h long cognitive tests. These tests are routinely employed in early AD diagnosis. The patient was free to interact with the doctor and the fNIRS-EEG probing did not impose any major physical constraints. For the EEG, a power spectrum density of the recordings is also reported. The high quality of the data for both fNIRS and EEG is visible with the presence of physiological signals and absence of relevant artifacts.

## 4. Discussion

An innovative fiberless multichannel fNIRS-EEG system was here described. The SiPM-based fNIRS component was constituted of 256 channels (16 bicolor LEDs and 16 SiPMs) with optical probing mechanically resembling EEG electrodes. All the required fNIRS electronic processing was hosted by a FPGA technology implementing a full digital lock-in technique for increased SNR and absence of crosstalk among channels and with the EEG component of the system. A benchtop characterization of the fNIRS component showed an end-to-end NEP of the optical instrumentation of 0.1–0.5 pW with a sampling frequency of 781.2 Hz (Figure 5). This result, considering the high sampling frequency used for the test and the physiological bandwidth of fNIRS that does not exceeds the fraction of Hz, is clearly an upper boundary value, depicting the ability of the system to acquire fNIRS signal at particularly large interoptode distances, also when accounting for the large SiPM photon harvesting capabilities. In fact, the system displayed a linearity of the response up to 70 mm interoptode distance on phantoms mimicking optical properties of biological tissues (Figure 6). When performing in vivo measurements, heart pulsations were visible in all the channels investigated (Figure 7). The visibility of a physiological pulsatile signal is essential in fNIRS signal quality evaluation since its magnitude is comparable with the hemodynamic oscillations induced by brain activity [39]. The EEG component was a modified version of a commercially available EEG system (g.HIamp, g.tec medical engineering) composed of 19 active electrodes. Both systems could be easily expanded up to 1024 channels for the fNIRS (32 time-multiplexed bicolor sources and 32 detectors), with a decrease in maximum measurement rate by a factor of two, i.e., 24 Hz, and up to 256 electrodes for the EEG components. The in vivo capabilities of the system and its excellent performance when evaluating brain activity were characterized through a battery of tasks eliciting activation in the motor, visual and frontal cortices (Figure 3, Figure 4, Figure 8 and Figure 9). Importantly, by exploiting the characteristics of controlaterality of the activation in the motor and visual cortices, neurovascular coupling was estimated through the synchronous EEG and fNIRS recordings (Figure 10). Finally, the feasibility of long-term ecological recordings with the system developed was demonstrated performing a ~1 h long measurement on one early AD patient in ambulatory settings during cognitive tests routinely performed for clinical diagnosis (Figure 11) [8].

The results presented here showed the capabilities of SiPMs for fiberless fNIRS imaging and their possibility to represent state-of-the-art detectors for wearable optical neuroimaging. In fact, these results have to be compared with current fiberless CW-fNIRS technology. Fiberless CW-fNIRS generally employs photodiodes for light detection. Photodiodes sensitivity is poor and, considering the average NIR light attenuation in the head (~1 decade/cm) [62], and possible absorption from hair, the systems are generally limited to forehead measures employing few sparse optodes at fixed and small source–detector distances (maximum of ~30 mm). These features limit the fiberless fNIRS field of view, localization power and depth investigation capabilities [63]. Thanks to the high sensitivity and large detection area of SiPMs, the optical component of the system presented here overcomes the current limitation of fiberless fNIRS technology, providing a sensitivity resembling that of fiber-based systems. Importantly, it is well known that the presence of hair can impair the optical recordings. However, this relevant fNIRS issue can be drastically dampened by the SiPMs’ large detection area. Although some light will be absorbed by hair, the large area of the SiPMs allows sensitivity to scalp regions where light passes through. In fact, this characteristic, coupled with the single photon sensitivity of SiPMs, allowed to perform in vivo measurements at large source detector distances without moving any hair. Moreover, through a simple ad-hoc arrangement of the optodes on the cap, it can be employed for sensitive high-density fNIRS-DOT. DOT configurations enable the acquisition of signals from short interoptode distance channels that can be used for regressing out scalp contamination in fNIRS analysis. Indeed, the fNIRS component developed can perform short channels measures. However, for the in vivo recordings reported in the manuscript, we preferred to use a sparse optode configuration in order to maximize the field of view of the recordings and sensitivity to different brain regions. Although fNIRS analysis using only long channels can be affected by scalp contamination, the statistical analysis was performed at a group level, where this effect is particularly small. In fact, since the strength and the phase of systemic physiological changes depends significantly on the given subject, the group-level analysis is the optimal approach to reduce false positives. Moreover, tasks protocols were designed to create a high contrast between the experimental and the baseline conditions in order to reduce the occurrence of false negatives [15]. This fiberless fNIRS-DOT solution can be easily coupled with EEG (Figure 1, Figure 2 and Figure 10) [1]. Scalp-located light sources and highly sensitive semiconductor detectors may play a key role in expanding fNIRS as a neuroimaging tool and in integrating fNIRS with EEG, in a cheap, lightweight, and portable technology that can assess electrical and hemodynamic brain activity as well as neurovascular coupling.

Ecological and long-term recordings of electrical and hemodynamic brain activity together with the evaluation of neurovascular coupling could allow diagnosis and monitoring of acute and chronic diseases that influence brain status and cerebrovascular health (e.g., epilepsy, stroke, vascular dementia, Alzheimer’s Disease) [8,64,65,66]. Such a neuroimaging tool can also be employed during intensive care and surgery [67,68,69,70,71,72]. Furthermore, the technology may be essential in rehabilitation protocols for studying brain plasticity and for implementing neurofeedback protocols based on brain computer interface approaches [73,74]. Future directions foresee the integration of SiPM-based fNIRS and dry EEG electrodes in a completely wireless communication technology in order to further optimize wearability, portability and ease of utilization of this multimodal brain monitoring technology.

## 5. Conclusions

A novel multichannel fNIRS-EEG system was developed. This device can answer the need of portable systems to accurately monitor electrical and hemodynamic brain activity in ecological and clinical settings. The fNIRS component relied on fiberless scalp-located optical technology based on SiPMs with mechanical properties of the optical probing resembling EEG electrodes. SiPMs provided high sensitivity and maximized photon harvesting. These characteristics, coupled with digital lock-in detection, allowed the system to outperform current fiberless fNIRS technology. Using motor, visual and cognitive tasks, we established that brain electrical and hemodynamic activity, as well their link, i.e., the neurovascular coupling, could be accurately retrieved. Ambulatory recordings on one patient with early AD demonstrated the portability of the device and the potential to provide useful clinical information through ecological measurements. Thanks to the SiPMs characteristics, fNIRS can be performed employing high density optical arrays with large interoptode distances delivering high spatial localization and depth investigation capabilities through DOT, paving the way to a widespread clinical employment of accurate fNIRS-EEG.
